# Thermophysical properties of polyethylene glycol oligomers *via* molecular dynamics simulations†

**DOI:** 10.1039/d4ra04898a

**Published:** 2024-09-03

**Authors:** Thi H. Ho, Tong Duy Hien, Øivind Wilhelmsen, Thuat T. Trinh

**Affiliations:** a Laboratory for Computational Physics, Institute for Computational Science and Artificial Intelligence, Van Lang University Ho Chi Minh City Vietnam thi.hohuynh@vlu.edu.vn; b Faculty of Mechanical – Electrical and Computer Engineering, School of Technology, Van Lang University Ho Chi Minh City Vietnam; c Faculty of Engineering, Vietnamese-German University (VGU) Thu Dau Mot City Binh Duong Province 75000 Vietnam; d Department of Chemistry, Porelab, Norwegian University of Science and Technology Trondheim Norway thuat.trinh@ntnu.no

## Abstract

Polyethylene glycol (PEG) is a versatile chemical with numerous applications in various fields, including biomedical research, pharmaceutical development, and industrial manufacturing. Molecular dynamics (MD) is a powerful tool for investigating the thermophysical properties of PEG molecules. In this study, we employ the General AMBER force field (GAFF) to perform MD simulations on various PEG oligomers, focusing on the calculation of density, self-diffusion coefficients, shear viscosity, and thermal conductivity. The results demonstrate excellent agreement with experimental data, where GAFF outperforms other force fields in reproducing thermophysical properties. For a PEG tetramer, the GAFF force field reproduces experimental data within 5% for the density, 5% for the diffusion coefficient, and 10% for the viscosity. In comparison, the OPLS force field displays significant deviations exceeding 80% for the diffusion coefficient and 400% for the viscosity. A detailed analysis of partial charge distributions and dihedral angles reveals that they significantly impact the structural behavior of PEG oligomers. The findings highlight the GAFF force field as one of the most accurate and reliable options for simulating systems with PEGs.

## Abstract 2

The thermophysical properties of the polyethylene glycol oligomer obtained by the AMBER force field are in excellent agreement with experimental data.

## Graphical abstract

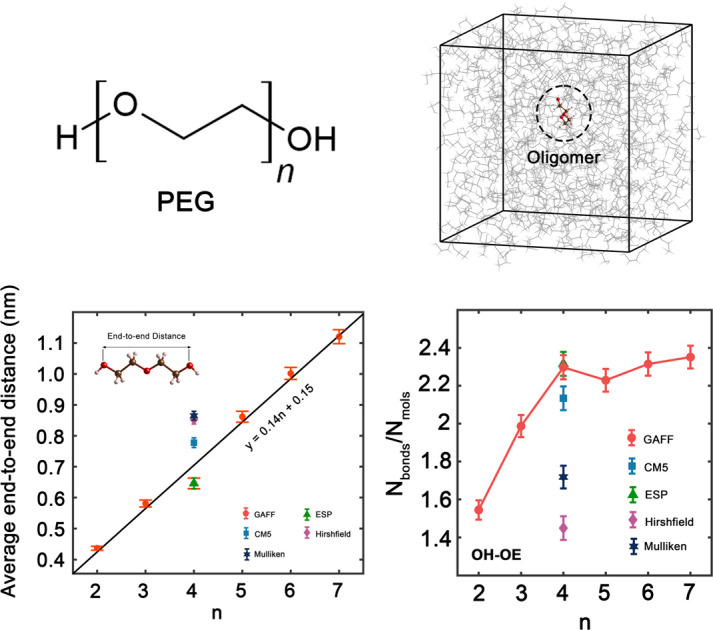

## Introduction

**Fig. 1:**
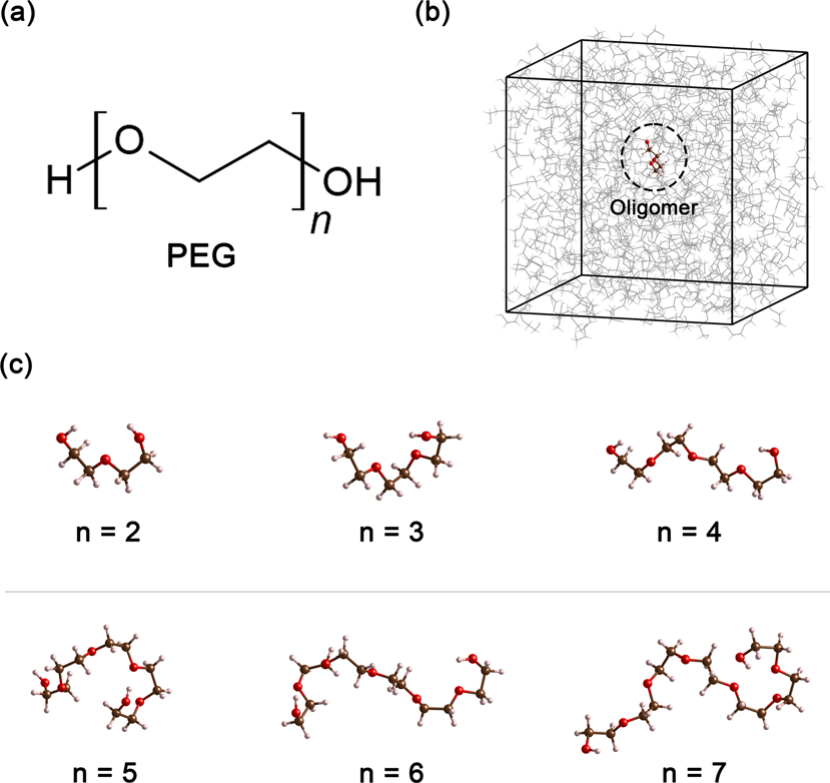
(a) Chemical formula of PEG200, where *n* signifies the number of repeating ethylene oxide units. (b) Cubic unit cell in MD simulation, containing an oligomer molecule at its center for presenting neat PEG oligomer with size *n* (c) snapshot of six oligomers obtained from the MD simulation, illustrating the increase in the number of repeating units, *n* from 2 to 7.

Polyethylene glycol (PEG), a compound of significant versatility, holds a pivotal role across diverse industries. Its chemical structure is represented as H–[O–CH_2_–CH_2_]_*n*_–OH, as depicted in Fig. 1(a). With an annual production order of approximately 500 000 tons,^1^ this compound has garnered considerable attention due to its wide-ranging uses in the chemical and healthcare industries.^2^ For example, in the medical field, PEG is often used as an excipient in pharmaceutical products, serving as a base for various dosage forms, including oral, topical, and parenteral medications.^3,4^ Its role in enhancing the solubility and stability of drugs makes it a crucial component in pharmaceutical products. In the cosmetic industry, PEG is known for its multiple functions, such as a humectant that helps retain moisture, a solvent that aids in the dissolution of other substances, or a softener that contributes to the texture and feel of cosmetic products.^5,6^ In the manufacturing sector, PEG is utilized for its properties as an anti-foaming agent and plasticizer, and in the production of ceramics.^7–9^ Furthermore, PEG can also function as a heat transfer medium^10^ or a chemical solvent^11^ when PEG is present as a major component in the system.

The vast applications of PEG can be attributed to its unique physical and chemical properties. Considering safety aspects, PEG is environmentally benign and non-toxic, possesses a low vapor pressure, thereby minimizing the risk of inhalation exposure.^12^ Its biodegradable nature further enhances its ecological compatibility, making it a preferred choice in green chemistry.^13^ In addition to its advantageous properties as a green chemical solvent, PEG is appealing due to its cost-effectiveness and superior solvent capabilities compared to ionic liquids for certain solutes.^14^ This enables its usage as a solvent for various mineral salts^15^ and also allows PEG to be used as a medium to fabricate metal organic frameworks (MOFs).^16,17^ Recently, the interest in PEG as a solvent for chemical synthesis and separations has increased, as shown by recent studies.^12,18,19^ This highlights the ongoing exploration of the potential for PEG in various fields and its importance in future scientific advancements.

PEG is commercially available as a polydisperse mixture, where the average molar weight of these mixtures is indicated by the product name. For example, PEG200 indicates that the average molar weight is approximately 200 g mol^−1^. In recent years, the physical properties of PEG, such as its density, velocity, self-diffusion coefficient, and thermal conductivity, have become the focus of extensive research. These properties are crucial for understanding the behavior of PEG in different applications. Numerous investigations have reported the densities^20–36^ viscosities,^20–24,28,30,32–34,37,38^ and thermal conductivities^39–44^ of PEG. However, a comprehensive understanding of these properties remains an ongoing subject for both experimental and theoretical studies. In experimental studies, the values of the thermophysical properties of neat (pure) PEG are typically obtained from extrapolations to zero content of water or other solvents.^11,38^ Consequently, these properties may vary from vendor to vendor and batch to batch for PEG, posing challenges for accurate determination of these properties for neat PEG.

Employing molecular dynamics (MD) simulations to calculate thermophysical properties is a powerful and widely used methodology, offering insights into the behavior of materials at the molecular level.^11,45–48^ However, the selection of appropriate force fields, which accurately represent the interatomic interactions in the system, poses a significant challenge. A review of the literature reveals that while there are numerous force fields available for glymes, which are structurally similar to PEGs, there are only a limited number of force fields specifically designed for PEGs.^49^ These include GAFF/AMBER,^50,51^ CGenFF/CHARMM,^52,53^ GROMOS,^54^ OPLS,^55^ Martini,^56^ and a specific force field from the Müller–Plathe group.^57^ These force-fields were frequently created and examined for aqueous solutions containing PEG, but not for neat PEG. Furthermore, each of these force fields has its own strengths and limitations, and the choice of force field can significantly impact the results from MD simulations.

Recently, Hoffmann *et al.*^45^ conducted MD simulations to evaluate the accuracy of available force fields for neat PEG200, focusing on replication of experimental properties, particularly the density and self-diffusion coefficients. Their findings show that there are discrepancies in the results between various force fields and simulation specifications, and suitable modifications to the force field could improve the simulation results. The authors claimed that OPLS is currently the best force field for simulating PEG oligomer properties.^45^ However, there remain significant differences between simulation results and experimental data.^45^ Therefore, further development and testing of force-fields for neat PEG is necessary to enhance the understanding of PEG and its suitability for different applications.

In this study, we investigate neat PEG oligomers by use of MD simulations with the General AMBER force field (GAFF). The accuracy of the GAFF force field is assessed by comparing the MD result to existing experimental data, focusing on density, self-diffusion coefficients, viscosity, and thermal conductivity. Our results demonstrate excellent agreement with the existing data from the literature for all ethylene glycol oligomers. Furthermore, we analyze the results from the MD simulations to delve deeper into the effects of bonding behavior, elucidating and interpreting the origins of changes in the radial distribution functions, as well as structural and thermophysical properties. In an attempt to improve the results, we adjust the electrical charge of the atoms. The modifications reveal strong correlations between the charge balance and the properties of PEG molecules.

The structure of the paper is as follows. The Methods section elucidates the details of the MD simulations and the analyses derived from the obtained MD trajectories. The Results and discussion section provides a summary of the MD simulation results and a comparison with the existing literature data. This is followed by a presentation of the results from the modifications of the GAFF force field by altering the electrical charge of atoms. Important structural measures, including average end-to-end distances, radii of gyration, hydrogen, bonding, and dihedral angle distribution were analyzed to find relationships between oligomer size and the properties of PEG oligomer. Finally, the Conclusions section encapsulates the key findings.

## Computational methods

### Simulation details

All simulations of neat ethylene glycol oligomers with *n* = 2–7, illustrated in Fig. 1(c), were performed using the large-scale atomic/molecular massively parallel simulator (LAMMPS) code.^58,59^ The simulation temperature was 328 K, which is in the middle range (298–358 K) of available experimental data.^37,38,60^ In this work, the GAFF^50,51^ force field was utilized to describe the molecular interactions.

For oligomers of PEG200, where no improper torsions are applied, the potential function is:1
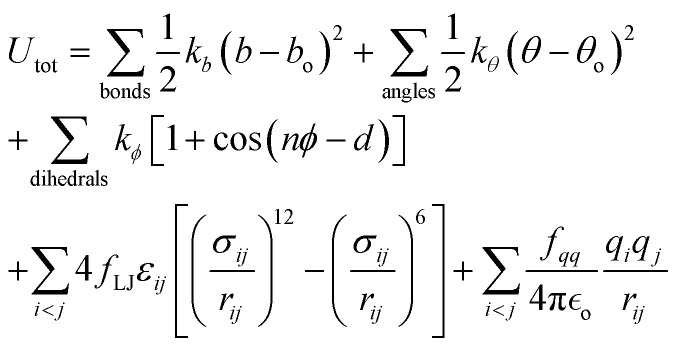
where bonds and angles are represented by simple harmonic oscillators with spring constants *k*_*b*_ and *k*_*θ*_ and equilibrium bond lengths and angles *b*_o_ and *θ*_o_, respectively. *r*_*ij*_ is the distance between atom *i* and *j* and the proper dihedrals are described by a cosine potential with energy barrier coefficient *k*_*ϕ*_, period *n*, torsional angle *ϕ*, and phase shift *d*.

The Lennard-Jones (LJ) interaction parameters contact distance *σ*, and well-depth *ε*, between two atoms are calculated according to geometric mean combination rules 
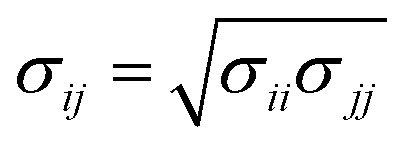
 and 
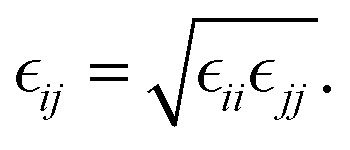
 The fudge factors *f*_LJ_ and *f*_*qq*_ for Lennard-Jones and Coulomb interactions, respectively, are zero for nonbonding interactions between 1–2 and 1–3 atom pairs, 0.5 for 1–4 pairs, and 1 for all other atom pairs. This means that the non-bonding interactions are only computed between atoms if they are located more than three covalent bonds apart or are present on different molecules. We present the non-bonding interactions between atom pairs in Table 1. The parameters for bonding interactions, which include bond stretching, angle bending, and dihedral torsion, are listed in Tables S1–S3 in the ESI.†

**Table 1:** Non-bonding parameters for Lennard-Jones potential used in simulations

Chemical group	Atom	*ε* (K)	*σ* (Å)	*q* (e)
Hydroxyl (–O–H)	O	105.85	3.07	−0.65
	H	0.00	0.00	0.42
Ether (–CH_2_–O–)	C	55.05	3.40	0.05
	H	7.90	2.47	0.09
	O	85.55	3.00	−0.34

For atomic charge modification, density functional theory (DFT) calculations were performed using the Gaussian 09 software suite.^61^ The B3LYP hybrid functional,^62–64^ in conjunction with the 6-311+G(d,p) basis set, was utilized to derive the atomic charges from four distinct charge models: CM5, Hirshfeld, Mulliken, and ESP. The general AMBER force field (GAFF) charge and atomic charges obtained from these models are presented in Table 2.

**Table 2:** Atomic charges across distinct charge models: GAFF, CM5, Hirshfeld, Mulliken, and ESP

Chemical group	Atom	GAFF	CM5	Hirshfield	Mulliken	ESP
Hydroxyl (–O–H)	O	−0.65	−0.46	−0.25	−0.32	−0.75
	H	0.42	0.34	0.17	0.24	0.44
Ether (–CH_2_–O–)	C	0.05	−0.06	0.01	−0.18	0.36
	H	0.09	0.1	0.4	0.14	−0.02
	O	−0.34	−0.28	−0.18	−0.21	−0.66

Neat PEG systems were prepared by randomly inserting a total of 250, 500 or 1000 PEG oligomer molecules into a cubic box using the Packmol code,^65^ schematically shown in Fig. 1(b). Following preparation, the systems were subjected to energy minimization using the conjugate gradient algorithm^66^ to eliminate high-energy contacts that were generated during system preparation. The minimization continued until the system reached a local energy minimum or until a maximum of 50 000 steps had elapsed. A distance of 1.4 nm was chosen for the cut-off of all non-bonding interactions, including electrostatics and LJ interactions. For the treatment of long-range electrostatic interactions, the Edward summation method was employed. During the energy minimization, no long-range analytic tail dispersion corrections for the energy and pressure were applied.

After the energy minimization, each system was simulated in the isothermal-isobaric (*NPT*) ensemble. The simulation temperature and pressure were set to 328 K and 1 atm, with a time step of 2 fs. Initial velocities were generated for each atom using a random number generator. The *NPT* simulations were conducted for 10 million time steps (20 ns), a duration sufficient for the system density to converge. In every time step of the simulation, all hydrogen bonds in the PEG oligomer molecules were constrained using the SHAKE algorithm.^67^ The numerical integration of Newton's equations of motion was performed using the Stoermer–Verlet time integration algorithm.^68^ The non-bonding interactions were treated similarly to the energy minimization process, which included a 1.4 nm cutoff distance and the long-range treatment of electrostatic interaction. The system temperature was regulated using the Nose–Hoover temperature thermostat, and pressure was controlled using the Nose–Hoover pressure barostat.

After the *NPT* simulation, the average density from the converged region was selected to scale the system size to the average volume. Next, simulations in the canonical ensemble (*NVT*) were conducted at a temperature of 328 K. All parameters from the preceding *NPT* simulation were retained, with the exception of pressure coupling, which was deactivated. Each simulation was set to run for 50 million time steps (equivalent to 100 ns), a duration that allows the system to achieve numerical convergence. In addition, for the thermal conductivity, the total MD time was doubled. A minimum of 5000 position frames were recorded, and the energy frames were recorded every 25 fs to accurately obtain self-diffusion, shear viscosities, and thermal conductivity *via* the Green–Kubo (GK) integral time decomposition method,^69^ which is further elaborated in the next subsection.

### Details about the analysis

All analysis was performed using modules available in the LAMMPS code or the OCTP plugin,^70^ a tool for on-the-fly calculation of transport properties of fluids with the order-n algorithm for LAMMPS. The density was obtained from *NPT* simulations, while self-diffusion coefficients, shear viscosity, radial distribution functions, hydrogen bonding numbers, and dihedral angle analyses were obtained from *NVT* simulations.

The density was calculated from averaging instantaneous densities over the specific time frames of the trajectory from the *NPT* simulation. The instantaneous density is computed as follows:2
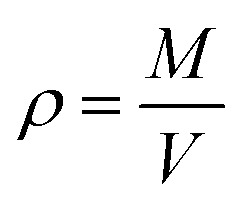
where *M* is the total mass of the system and *V* is the instantaneous volume of the simulation box at some point in the trajectory. The average density of a system 〈*ρ*〉 is then obtained by averaging the instantaneous densities over the trajectory region for which the density has reached equilibrium.

Self-diffusion coefficients were obtained from the *NVT* simulation using the Einstein-relation^71^ as follows:3
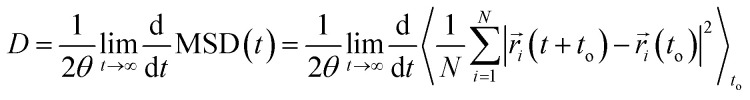
where 〈…〉_*t*_o__ refers to averaging over multiple time origins *t*_o_, which were increased in increments of 25 ps, *θ* = 3 for a three-dimensional system, and MSD(*t*) is the mean squared displacement at time *t* over all atom N. The value of *D* was obtained using the OCTP plugin.^70^

The analytic correction by Yeh and Hummer was used to calculate the self-diffusion coefficient at infinite box size:^72^4
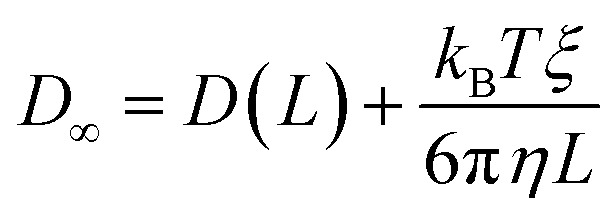
where *k*_B_ is the Boltzmann constant, *T* is the temperature, *ξ* = 2.837298, and *η* is the calculated shear viscosity.

The shear viscosity (*η*) was computed from the time integral over the auto-correlation function of the off-diagonal components of the pressure tensor (*P*^os^_*αβ*_):^70^5
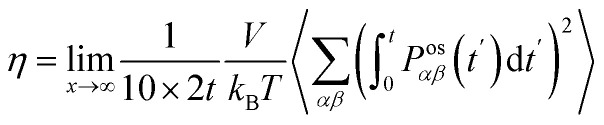
where6
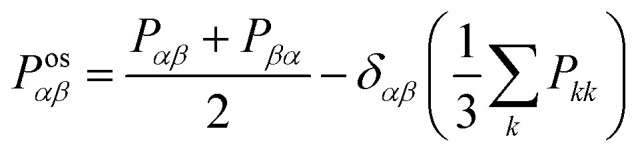
and *δ*_*αβ*_ is the Kronecker delta. The last term, *i.e.* one-third of the invariant trace of the pressure tensor, equals the instantaneous kinetic pressure of the system (*p*). The contribution of the diagonal components of the pressure tensor to the shear viscosity in eqn (5) is 4/3. Therefore, the contribution of all 9 components of the trace-less pressure tensor results in the factor 10 in the denominator of eqn (5). The value of *η* was computed using the OCTP plugin.^70^

The thermal conductivity (*λ*) was calculated from the components of the energy current/heat flux (*J*_*α*_) as follows:^70^7
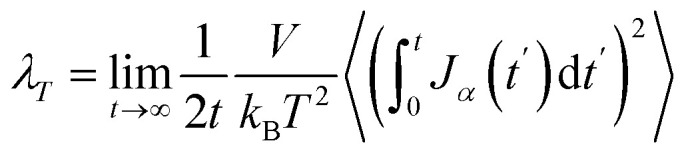
the total heat flux consists of two parts: the kinetic heat flux (*J*_kinetic_) and the potential heat flux (*J*_potential_). The total heat flux is calculated from:^70^8
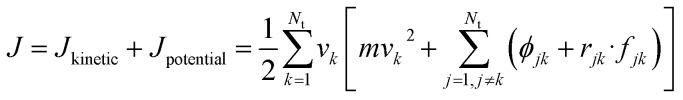
where *N*_t_ is the total number of atoms in the system. *v*_*k*_ is the velocity vector of atom *i*. *ϕ*_*jk*_, *r*_*jk*_, and *f*_*jk*_ are the interaction potential, distance, and force between the two atoms *j* and *k*. The value of *λ* was computed using the OCTP plugin.^70^

The radial distribution function (RDF), or pair correlation function between particles A and B with particle A as the central reference atom is computed as follows,9
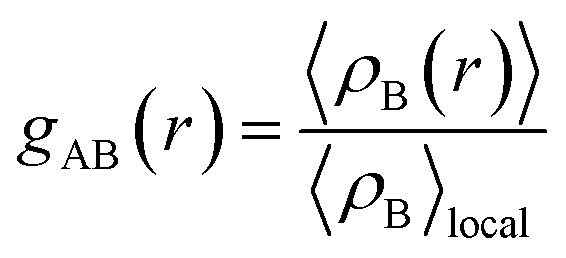
where 〈*ρ*_B_(*r*)〉 presents the average particle density of B at a distance *r* away from particle A, and 〈*ρ*_B_〉_local_ is the particle density of B averaged over all spheres centered upon particle A with a radius half the box length. RDFs between two types of particles A and B were calculated using the analysis extension in the VMD program.^73^

**Fig. 2:**
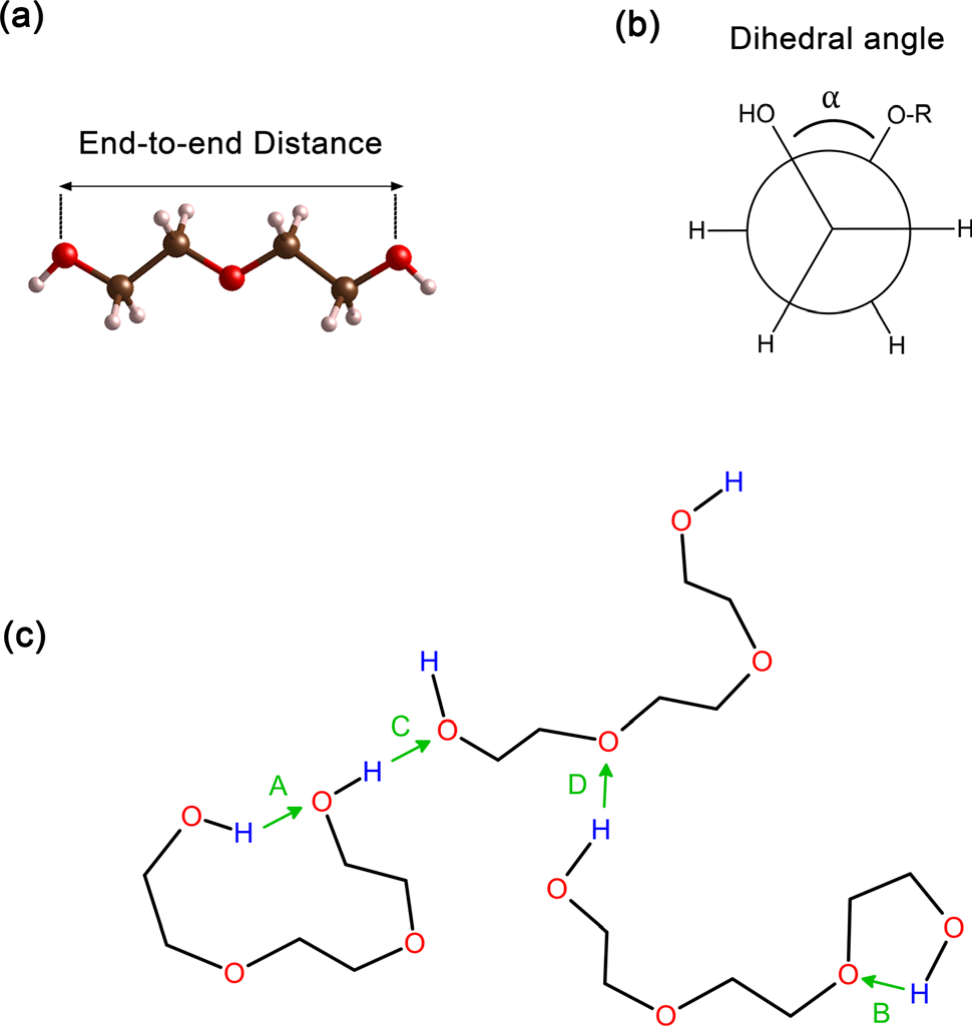
(a) The distance between the oxygen atoms of the terminal hydroxy end groups of oligomers used as a measure for the end-to-end distance. (b) Illustration of the proper dihedral angle. (c) Potential hydrogen bonding interactions: (A) represents intramolecular hydrogen bonding occurring between hydroxy end groups (OH–OH), (B) shows intramolecular hydrogen bonding between a hydroxy end group and an ether oxygen atom (OH–OE), (C and D) are analogous to (A and B) respectively, but they occur between two distinct molecules, signifying intermolecular hydrogen bonding.

In order to obtain comprehensive understanding of the size and compactness of the PEG structure, we will analyze the average end-to-end distances and average radii of gyration. These statistical measures provide valuable insights into the physical dimensions and spatial configuration of the PEG molecule. The end-to-end distance of a PEG molecule was calculated as the straight-line distance between two terminal hydroxy oxygen atoms, as shown in Fig. 2(a). The average end-to-end distance is then obtained by averaging distances over all PEG molecules and over time in the MD trajectory. The radius of gyration was calculated as the root mean square distance of the atoms of the PEG chain from its center of mass,10
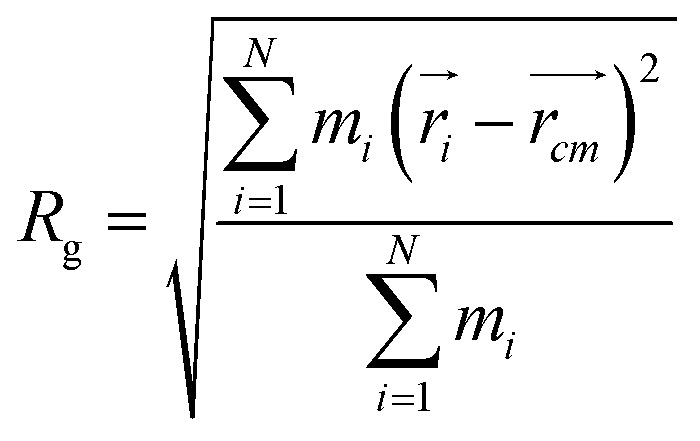
where 
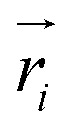
 and 
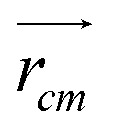
 are position vector of atom *i* and center of mass of the polymer chain, respectively, and *m*_*i*_ is the mass of atom *i*. The average radius of gyration is then obtained by averaging radii over all PEG chains and over time in the MD trajectory.

In addition, the distribution of proper dihedral angles was calculated to assess the conformational flexibility of molecules and to validate the accuracy of the force fields used in MD simulations. The dihedral angle *α* is defined as the angle formed by (HO)–C–C–(OR) segment, as shown in Fig. 2(b).

Hydrogen bonds serve as a crucial non-covalent structural force within molecular systems. In order to delve deeper into the strength and characteristics of the hydrogen bonding in the PEG structure, we performed an analysis of hydrogen bonding. The different types of hydrogen bonds are illustrated in Fig. 2(c). The H bonds plugin in the VMD program^73^ was utilized to calculate the number of hydrogen bonds at instantaneous points in time. Hydrogen bonding donor and acceptor groups were tabulated to determine the number of hydrogen bonds between a donor atom (D) and an acceptor atom (A) at any given point in time. The number of hydrogen bonds at an instantaneous point in time was then defined as the number of A–D pairs that met the criteria of the A–D distance being ≤0.35 nm and the angle A–H–D being ≤120°. The average number of hydrogen bonds was subsequently computed by averaging all the instantaneous hydrogen bond numbers over all trajectory frames.

## Results and discussion

### Thermophysical properties

Table 3 compares the calculated and experimental values for the thermophysical properties of PEG oligomers at *T* = 328 K. The calculated properties, which include the density, the self-diffusion coefficient (*D*), the viscosity (*η*), the product *Dη*, and the thermal conductivity (*λ*), were obtained using the GAFF force field. Each of these properties were obtained as a function of the oligomer size, denoted by *n*, which was varied from 2 to 7. Overall, the GAFF force field has a good ability to replicate the trends of the experimental data in the literature.

**Table 3:** Comparison with experimental data for the thermophysical properties at *T* = 328 K for ethylene glycol oligomers, HO(CH_2_CH_2_O)_*n*_H, simulated with the GAFF force field for oligomer sizes *n* = 2–7 and a system size of *N* = 250 molecules. (%) denotes the percentage difference between experimental and calculated results

*N* = 250	*n* = 2	*n* = 3	*n* = 4	*n* = 5	*n* = 6	*n* = 7
**Density in kg m** ^**−**3^						
Exp^37^	1091.7 ± 0.1	1097.1 ± 0.1	1096.0 ± 0.1	1097.4 ± 0.2	1099.3 ± 0.9	1097.0 ± 1.0
This work	1143.1 ± 0.2	1137.7 ± 0.2	1143.3 ± 0.1	1145.1 ± 0.1	1147.5 ± 0.1	1149.9 ± 0.1
(%)	5%	4%	4%	4%	4%	5%

**Self-diffusion coefficient, *D*, in 10** ^**−11**^ **m** ^**2**^ **s** ^**−1**^						
Exp^37^	17.4 ± 0.5	12.5 ± 0.1	10.0 ± 1.0	8.6 ± 0.1	6.6 ± 0.2	5.7 ± 0.1
This work	20.7 ± 0.2	17.2 ± 0.4	9.6 ± 0.2	7.1 ± 0.1	5.7 ± 0.7	4.4 ± 0.2
(%)	19%	38%	4%	17%	14%	23%

**Shear viscosity *η*, in mPa s**						
Exp^37^	9.0 ± 0.3	11.2 ± 0.2	13.2 ± 0.3	15.8 ± 0.1	18.3 ± 0.4	20.6 ± 0.5
This work	7.3 ± 0.5	8.0 ± 1.0	14.4 ± 0.6	17.9 ± 2.0	19.9 ± 2.0	20.3 ± 2.2
(%)	19%	29%	9%	13%	9%	2%

***Dη* (10** ^**−14**^ **N)**						
Exp^37^	156.6	140.0	132.0	135.9	120.8	117.4
This work	151.1	137.6	138.2	127.1	113.4	89.3
(%)	4%	2%	5%	7%	6%	24%

**Thermal conductivity *λ* (W (m** ^**−1**^ **K** ^**−1**^ **))**						
Exp (mixture)^44^	0.191 ± 0.003	0.191 ± 0.003	0.191 ± 0.003	0.191 ± 0.003	0.191 ± 0.003	0.191 ± 0.003
This work	0.233 ± 0.014	0.228 ± 0.021	0.208 ± 0.010	0.205 ± 0.003	0.219 ± 0.010	0.193 ± 0.022
(%)	22%	19%	9%	7%	15%	1%

With a number of molecules of *N* = 250, the deviations between simulated and experimental values of shear viscosity and thermal conductivity show a striking trend. As the oligomer size increases from *n* = 2 to *n* = 7, the deviation decreases significantly. In particular, the deviation for small oligomers (*n* = 2) is around 20%, while for larger oligomers (*n* = 7), it drops to 1–2% (Table 3). This suggests that as the oligomer size grows, the simulations become increasingly accurate in predicting these thermophysical properties. The situation is different for the density and self-diffusion coefficient, where the deviations with respect to experimental data are relatively insensitive to oligomer size. The deviation for density remains around 5% across all oligomer sizes (*n* = 2–7), indicating that the simulations accurately capture the density for all considered oligomer sizes. Similarly, the self-diffusion coefficient shows a consistent deviation of approximately 20%, suggesting that this property is also relatively unaffected by a change in oligomer size.

For an oligomer size of *n* = 4, the calculated results exhibit good agreements with the experimental data, highlighting the accuracy of the force field for this particular molecular configuration. A closer look at the self-diffusion coefficients reveals a noticeable decrease as *n* increases, suggesting a decrease in molecular mobility for larger oligomers. This trend aligns with the anticipated increase in molecular interactions and entanglements as the chain length grows. Conversely, the viscosities exhibit an inverse relationship, with values increasing with oligomer size. This pattern implies a direct correlation between molecular size and the viscous resistance in the system. The calculated values for the product of *Dη* and thermal conductivity *λ* also provide useful information. *Dη* serves as an indicator of the system's dynamic behavior, while *λ* reflects the ability to conduct heat.

Furthermore, the finite-size effects^70^ for self-diffusion coefficients were taken into account to understand the influence of the simulation box size on the computed results. The simulation results are summarized in Table 4, including density, self-diffusion coefficient *D*, interpolated *D*_∞_ using eqn (4), shear viscosity *η*, the product *Dη*, and the thermal conductivity *λ* for different system sizes (250, 500, and 100 PEG molecules) at *n* = 2. Overall, the finite-size effects do not significantly influence the simulated thermophysical properties. According to the Stokes–Einstein relation presented in eqn (4), *D*_∞_ remains nearly unchanged beyond the system sizes considered in this work. This observation indicates that finite-size effects do not strongly influence the simulated thermophysical properties for this oligomer.

**Table 4:** Finite-size effects on the thermophysical properties of different system sizes (*N* = 250, *N* = 500, and *N* = 1000 molecules) with an oligomer size of *n* = 2. The percentage differences between experimental data and calculated results are also presented

Properties (*n* = 2)	Exp.	*N* = 250	*N* = 500	*N* = 1000
Density in kg m^−3^	1091.7 ± 0.1 (ref. 37)	1143.1 ± 0.2	1143.0 ± 0.1	1143.2 ± 0.1
		(5%)	(5%)	(5%)
Self-diffusion coefficient, *D*, in 10^−11^ m^2^ s^−1^	17.4 ± 0.5 (ref. 37)	20.7 ± 0.2	21.0 ± 0.2	20.6 ± 0.1
		(19%)	(21%)	(18%)
*D*_∞_	—	21.0	21.2	20.8
Viscosities *η*, in mPa s	9.0 ± 0.3 (ref. 37)	7.3 ± 0.5	7.2 ± 0.6	7.1 ± 0.3
		(19%)	(20%)	(21%)
*Dη* (10^−14^ N)	156.6 (ref. 37)	151.1	151.2	146.3
		(4%)	(4%)	(7%)
Thermal conductivity *λ* (W (m^−1^ K^−1^))	0.191 ± 0.003 (ref. 44)	0.233 ± 0.014	0.250 ± 0.018	0.245 ± 0.010
		(22%)	(31%)	(28%)

Table 5 compares the experimental and simulated properties of tetraethylene glycol (TeEG) at 328 K with different force fields. The GAFF force field simulations reported in this work demonstrate good agreement with experimental values for various thermophysical properties. Specifically, the simulated density shows a small deviation of approximately 4% from the experimental value. Similarly, the self-diffusion coefficient (*D*) and viscosity (*η*) exhibit deviations of 4% and 9%, respectively, from their corresponding experimental values. Notably, the product of these two properties, *Dη*, also shows a deviation of around 5%. These results indicate that the GAFF force field simulation is capable of accurately predicting various thermophysical properties of the PEG tetramer.

**Table 5:** Comparisons of experimental and simulated properties of tetraethylene glycol at 328 K: densities, viscosities, and self-diffusion coefficients with various force fields. The percentages in the parentheses represent the difference between experimental data and results from the simulations

Force field	Density (kg m^−3^)	Self-diffusion coefficient *D* (10^−11^ m^2^ s^−1^)	Viscosities *η* (mPa)	*Dη* (10^−14^ N)
GAFF (this work)	1143.3 (4%)	9.6 (4%)	14.4 (9%)	138.5 (5%)
Exp^37^	1096.0	10.0	13.2	132.0
GROMOS^45^	1034.2 (6%)	5.9 (41%)	23.9 (81%)	141.0 (7%)
CGenFF^45^	1084.9 (1%)	2.7 (73%)	50.4 (282%)	136.1 (3%)
CZMP^45^	883.7 (19%)	35.1 (251%)	3.1 (77%)	108.8 (18%)
Martini^45^	951.6 (13%)	86.9 (769%)	2.4 (82%)	208.6 (58%)
OPLS^45^	1103.4 (<1%)	1.56 (84%)	65.5 (396%)	102.2 (23%)

We have compared our results to those reported earlier using various force fields such as GROMOS, CGenFF, CZMP, Martini, and OPLS in capturing the properties of PEG oligomer at *n* = 4. For the density, both CGenFF and OPLS are in excellent agreement with experimental values, with errors below 1% (Table 5). However, the excellent performance in predicting the density comes at the cost of significant deviations in other properties. In particular, CGenFF and OPLS exhibit large errors of around 80% for the self-diffusion coefficient and 300–400% for the viscosity, indicating that these force fields are not well-suited for predicting transport properties.

For the self-diffusion coefficient, the results using the GAFF force field show the lowest deviation from experimental data, with an error of only 4%. This is significantly better than other force fields, where GROMOS reports a deviation of 41% and MARTINI shows a huge deviation of nearly 800%, making it the least suitable force field in this regard. For the viscosity, the results with the GAFF force field show a significant improvement with respect to other force fields, with an error of only 9%. This is followed by the CZMP model, which reports a deviation of 77%, while OPLS shows the largest deviation of almost 400% (Table 5).

It is also interesting to analyse the product *Dη*, however, it can be missleading of not examined carefully. For instance, the CGenFF force field reported an impressive 3% deviation for the product *Dη*, but upon closer inspection, it is evident that *D* is underestimated by 73%, while the viscosity *η* is overestimated by 282%. Similarly, the GROMOS force field shows an acceptable 7% deviation, but this is also despite of large errors in *D* and *η*. Therefore, we recommend that this indicator should be thoroughly analyzed to avoid incorrect conclusions about the suitability of a force field to reproduce transport properties from experiments.

### Atomic charge modifications to the GAFF force field

In an effort to improve the agreement with experimental data, we have modified the GAFF force field by adjusting the GAFF charge. To this end, four different charge models were evaluated: CM5, Hirshfield, Mulliken, and ESP. Table 2 provides a detailed overview of the atomic charges for each atom, carbon (C), oxygen (O), and hydrogen (H), within the hydroxyl and ether chemical groups of oligomers. In the hydroxyl group, the charge of the O atom varies much with the model, with the GAFF model suggesting a negative charge of −0.65, while the Hirshfeld model has −0.25. The charge of the H atom also shows a considerable variation, with the highest positive charge being 0.44 in the ESP model, and the lowest positive charge being 0.17. In the ether group, similar to the hydroxyl group, the atomic charges of the C, H, and O atoms vary much with the model. The atomic charge of the C atoms ranges from a positive value of 0.36 for the ESP model to a negative value of −0.18 for the Mulliken charge model. Meanwhile, the C atoms are almost neutral for the GAFF, CM5, and Hirshfeld models. The charge models seem to agree on the atomic charges of the H atoms, except for the Hirshfeld charge model that has a value of 0.4. The atomic charges of O atoms vary much, with the highest negative value being −0.66 for the ESP model to the smallest value of −0.18 for the Hirshfeld model.

Table 6 compares the simulated and experimental values for the thermophysical properties of PEG200 at *T* = 328 *K*. The properties are simulated using the GAFF force field, both in its original form and with charge modifications. The experimental density of 1096.0 kg m^−3^ aligns closely with the GAFF force field when the Mulliken charge modification is applied, yielding a value of 1096.3 kg m^−3^. However, the GAFF, GAFF, CM5, and ESP models slightly overestimate the density, while the Hirshfeld model slightly underestimates it. In addition, the self-diffusion coefficient, experimentally determined to be 16.8 × 10^−11^ m^2^ s^−1^, is well approximated only when the GAFF charge model is used. It is overestimated with the CM5, Hirshfeld, and Mulliken charge models, and underestimated with the ESP charge model. The viscosity, experimentally measured to be 9.25 mPa s, is accurately approximated with both the GAFF and ESP charge models. Lastly, the thermal conductivity, with an experimental value of 0.21 W (m^−1^ K^−1^), aligns perfectly with the ESP charge modification, while the Hirshfeld modification leads to an underestimation. Overall, the CM5 and Mulliken charge models enhance the agreement with experimental data for the density. However, other charge modifications do not improve the result for the self-diffusion coefficient and the viscosity. The thermal conductivity remains nearly unchanged with the charge modifications.

**Table 6:** Comparison of thermophysical properties for different charge modifications for tetraethylene at 328 K. The percentage in parentheses denotes the difference between experimental data and calculated results

Properties/charge	Exp.	GAFF	CM5	Hirshfield	Mulliken	ESP
Density, kg m^−3^	1096.0 ± 0.1	1143.3 ± 0.1	1107.2 ± 0.1	1047.6 ± 0.1	1096.3 ± 0.1	1143.2 ± 0.2
		(4%)	(1%)	(4%)	(<1%)	(4%)
Self-diffusion coefficient, *D*, in 10^−11^ m^2^ s^−1^	10.0 ± 1.0	9.6 ± 0.2	28.0 ± 0.9	68.1 ± 0.4	36.6 ± 0.7	9.8 ± 0.1
		(4%)	(180%)	(581%)	(266%)	(2%)
Viscosity *η*, in mPa s	13.2 ± 0.3	14.4 ± 0.6	4.1 ± 0.5	1.7 ± 0.1	3.4 ± 0.4	12.8 ± 0.9
		(9%)	(69%)	(87%)	(74%)	(3%)
*Dη* (10^−14^ N)	132.0	138.2	114.8	115.8	124.4	125.4
		(5%)	(13%)	(12%)	(6%)	(5%)
Thermal conductivity *λ* (W (m^−1^ K^−1^))	0.191 ± 0.003	0.208 ± 0.010	0.185 ± 0.016	0.162 ± 0.008	0.168 ± 0.008	0.208 ± 0.007
		(9%)	(3%)	(15%)	(12%)	(9%)

Interestingly, we observe that the hydroxyl group exhibits a less negative charge, whereas the ether group tends to be more positive, especially for the Hirshfield and Mulliken charge models. This could lead to a reduction in density, aligning it more closely with experimental values. However, such variations may cause an overestimation of the self-diffusion coefficient and an underestimation of viscosity, as shown in Table 6. This analysis highlights the critical impact of atomic charge on determining properties of PEG oligomers. In the next sections, we analyze how charge modifications affect the simulation results.

It is crucial to acknowledge that altering atomic charges fundamentally modifies the underlying force fields. Each force field employs a default method for determining atomic charges. For example, GAFF utilizes the restrained electrostatic potential (RESP) approach. By adjusting charge assignment methods, we can achieve better agreement with experimental data for certain properties; however, this customization may also impact other properties. As demonstrated in our work, modifying atomic charges did not lead to improved thermophysical properties of PEG oligomers.

### Structural properties of PEG

**Fig. 3:**
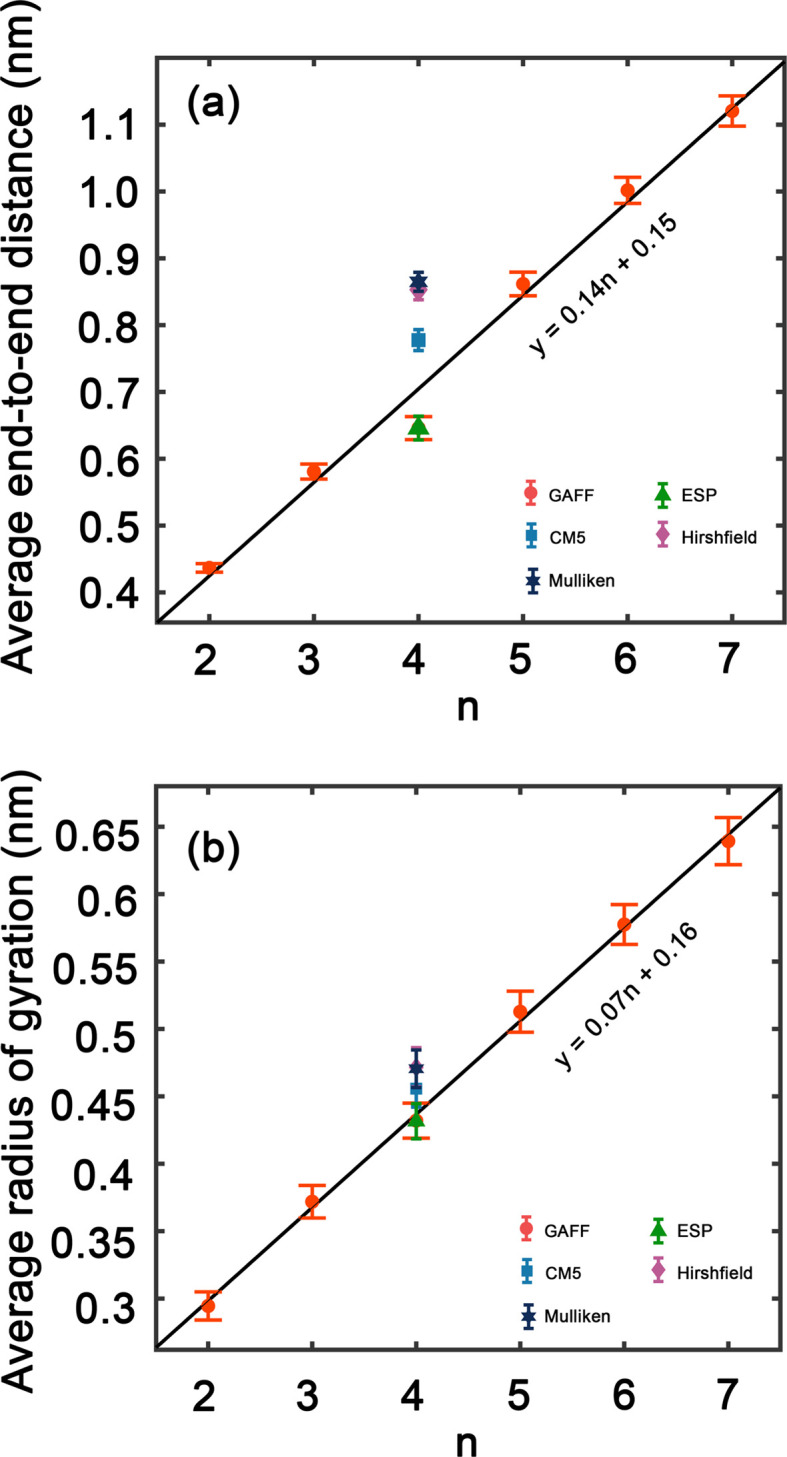
(a) Average end-to-end distance of ethylene glycol oligomers of hydroxy oxygen atoms and (b) average radii of gyration of ethylene glycol oligomers, ≤120° in PEG200 with included standard deviations. The values from different charge models are included for comparison. Solid lines are linear fits.

In order to improve the understanding of the dimensions and compactness of the PEG structure, we focus the analysis on oligomers with the sizes *n* = 2–7. Two key statistical measures will be discussed: the average end-to-end distances and the average radii of gyration, as depicted in Fig. 3. Fig. 3(a) reveals a linear correlation between the end-to-end distance of a PEG molecule and the oligomer size *n*. In Fig. 3(b), we observe a close to linear relationship between the radius of gyration and *n*, indicating a decrease in the compactness of the PEG molecule with an increase in *n*. An intriguing observation is the increase in standard deviation with *n*, which can be rationalized by the expansion of the structural configuration space as the length of the ethylene glycol oligomer increases. Large variations of average end-to-end distance of different charge models indicate a significant impact on the structural compactness of PEG molecules, giving insight into the differences in the predicted properties of PEG molecules shown in Table 6.

By comparing the behavior of PEG oligomer using GAFF force field in this work and the OPLS obtained by Hoffmann *et al.*,^45^ we observe a consistent trend in the average end-to-end distance and radius of gyration as a function of oligomer size. As the oligomer size increases, both properties exhibit a linear rise, which is indicative of the elongation and swelling of the polymer chain. The GAFF force field predicts a slightly lower average end-to-end distance for PEG oligomers compared to OPLS, suggesting that GAFF may describe the system with slightly stiffer chains. This discrepancy could be attributed to the inherent characteristics and parameterizations of each force field, which may influence their respective representations of chain stiffness and conformational behavior.

**Fig. 4:**
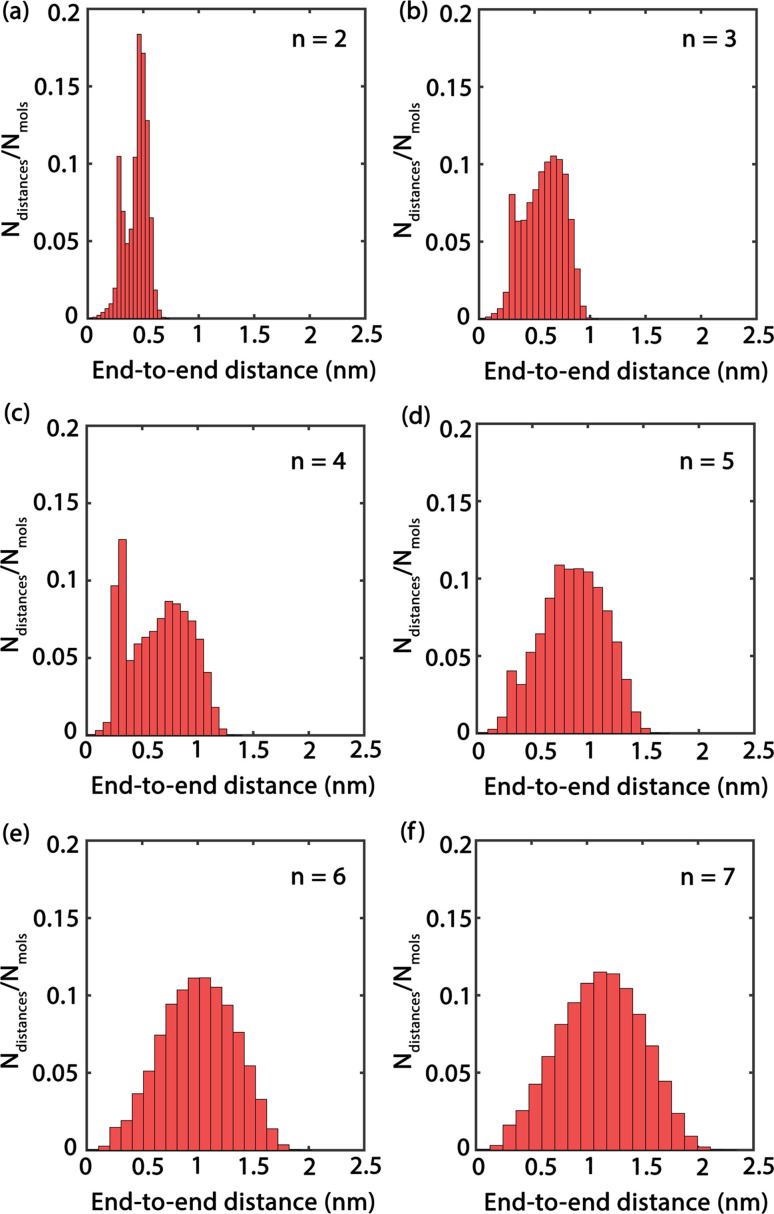
(a)–(f) Histograms of end-to-end distances with various oligomer sizes *n* = 2–7.

Fig. 4 presents histograms showing the end-to-end distances of PEG oligomers. A clear trend can be observed, where the histograms progressively shift towards right and broaden as *n* increases, indicating a correlation between oligomer size and spatial conformation. Specifically, the histogram for *n* = 2 in Fig. 4(a) reveals a large peak with a confined distribution, signifying the restricted conformational flexibility of the dimer and its tendency towards a compact structure. In contrast, the histogram for the trimer *n* = 3, shown in Fig. 4(b), displays a slight expansion and rightward shift of the peak, reflecting an increased average end-to-end distance and a broader molecular conformation. This pattern of broadening distribution persists for larger oligomer sizes *n* = 4 to *n* = 7, demonstrating the increased conformational diversity by the elongated PEG molecules that span from densely packed to extensively stretched structures.

**Fig. 5:**
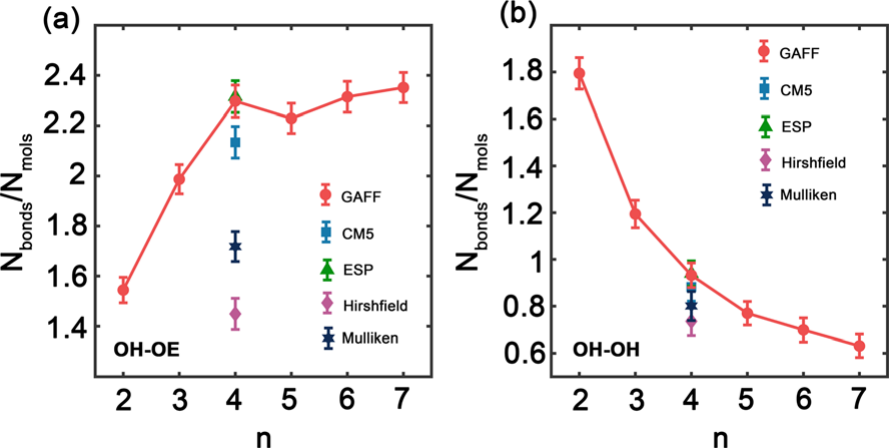
Number of hydrogen bonds between hydroxy hydrogen and (a) ether oxygen (OH–OE) and (b) hydroxy oxygen (OH–OH) per PEG molecule as function of oligomer size *n* using the original and charge modified GAFF force field.

Hydrogen bonds are fundamental aspects of the intermolecular forces in PEG. Hence, to analyze how the GAFF force field and its charge modifications characterize these interactions in PEG simulations is crucial. Such investigations will shed light on the implications of hydrogen bonding for the thermophysical properties observed for PEG oligomers, presented in Tables 3 and 6. Fig. 5 illustrates the variation in the number of hydrogen bonds involving hydroxy hydrogen with ether oxygen (OH–OE) and hydroxy oxygen (OH–OH) per PEG molecule, across different oligomer sizes *n*, as obtained by both the original and the charge-modified GAFF force fields.

Fig. 5(a) illustrates a significant increase in the number of OH–OE hydrogen bonds up to an oligomer size of *n* = 4. Beyond this size, the trend continues to rise but at a slower rate. In contrast, Fig. 5(b) shows a decrease in the number of OH–OH hydrogen bonds, with a significant drop observed after *n* = 2. Different charge modifications, namely CM5, ESP, Hirshfield, and Mulliken, within the GAFF force field, markedly influence the number of hydrogen bonds. These variations in hydrogen bonding play a crucial role in determining the properties of PEG oligomers, as detailed in Table 6. The more pronounced variation in OH–OE hydrogen bonds compared to OH–OH bonds suggests that interactions involving hydroxy hydrogen and ether oxygen are more significant in determining the properties of PEG.

In addition to our own simulations, we compare the hydrogen bond interaction results obtained using GAFF and OPLS force fields with those reported in the literature.^45^ For OH–OE interactions, both GAFF and OPLS demonstrate an initial increase in hydrogen bonding with rising oligomer size. Nevertheless, the rate of this increase differs between the two force fields. GAFF exhibits a more gradual peak at *n* = 4, followed by a slower and continuous rise, whereas OPLS displays a steeper ascent throughout the entire range of oligomer sizes.^45^ This difference suggests that the two force fields may describe the OH–OE hydrogen bonding behavior with varying degrees of accuracy or specificity.

In contrast, for OH–OH interactions, GAFF and OPLS predict significantly different rates of hydrogen bond formation as a function of oligomer size. GAFF depicts a relatively steep trend compared to OPLS,^45^ indicating that fewer hydrogen bonds are formed between hydroxy oxygen atoms with increasing oligomer size. This disparity underscores the potential discrepancies in representing the intramolecular hydrogen bonding within PEG oligomers when using different force fields.

**Fig. 6:**
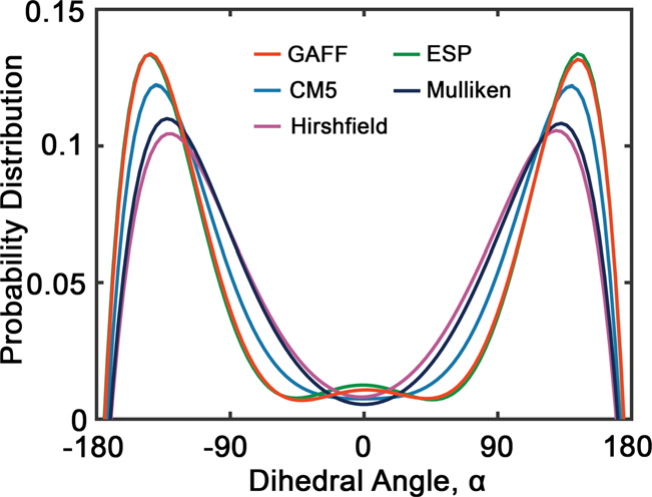
Probability distribution of dihedral angles (HO)–C–C–O–(OR) (see Fig. 2(b)) in tetraethylene glycol. Red, blue, purple, green and dark blue lines represent GAFF, CM5, Hirshfield, ESP, and Mulliken charges, respectively.

Fig. 6 presents the probability distributions of dihedral angles (HO)–C–C–O–(OR) in tetraethylene glycol (TeEG), using five different charge models: GAFF, CM5, Hirshfield, ESP, and Mulliken. Each curve exhibits a bimodal distribution with prominent peaks around ±140°, indicating that these angles are energetically favorable. Notably, the GAFF and ESP models display the largest peaks, suggesting that these charge assignments favor conformations with dihedral angles close to ±140° more strongly than the other models. In contrast, the CM5, Hirshfield, and Mulliken models show slightly smaller peaks, indicating a more moderate preference for these angles and allowing for smaller dihedral angles. This variation among the models underscores the sensitivity of dihedral angle distributions to the charge modification, reflecting how different electrostatic interactions can influence molecular conformations and, consequently, the thermophysical properties of the PEG oligomers.

Upon comparing the results obtained using GAFF and OPLS^45^ force fields, we observe several similarities and differences in their predictions. Both force fields predict similar preferred dihedral angles, as evidenced by the presence of two uphill peaks in their respective distributions. However, a notable difference between GAFF and OPLS lies in the positions of these peaks. For example, GAFF predicts peak positions approximately at ±140°, while OPLS suggests peak positions around ±60°.^45^ This discrepancy could imply variations in the predicted energy barriers or conformational flexibility between the two models.

**Fig. 7:**
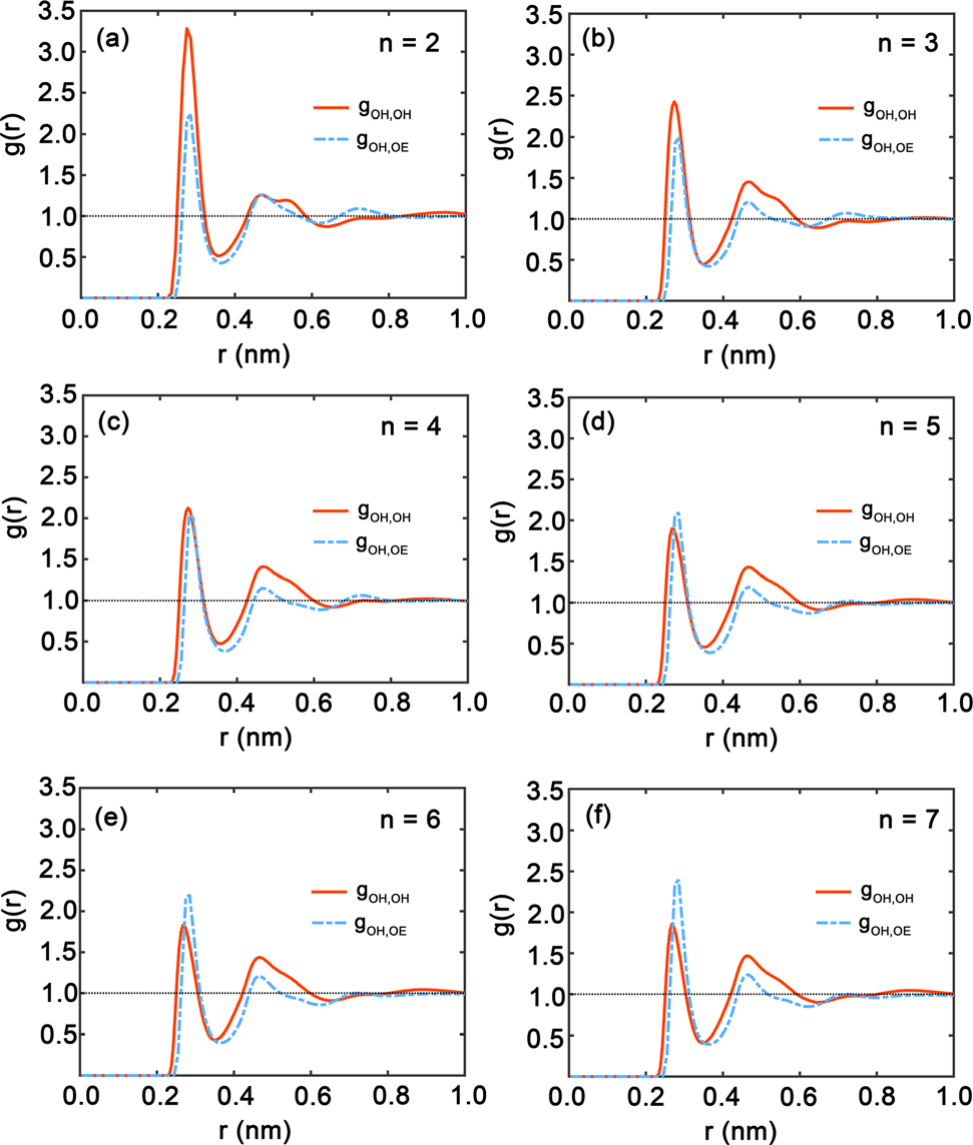
(a)–(f) Radial distribution functions (RDFs) between hydroxy oxygen and hydroxy oxygen (red) and ether oxygen (blue) for each oligomer obtained from simulating PEG oligomers at 328 K with the GAFF force field with *n* = 2–7.

Fig. 7 presents the radial distribution functions (RDFs), offering a statistical view of the spatial relationships between hydroxy and ether oxygen atoms within PEG oligomers. The RDFs are depicted by red and blue curves, representing hydroxy–hydroxy and hydroxy–ether oxygen pairings, respectively. A clear trend is observed as the oligomer length extends from *n* = 2 to *n* = 7; the peaks in the RDFs shift in position and grow in intensity, indicating a shift in atomic interactions and spatial configurations. Here, both first peaks in Fig. 7 are near 0.3 nm. The shapes of the *g*_OH,OH_ and *g*_OH,OE_ at distances exceeding 0.5 nm do not show significant difference. In particular, Fig. 7(a) for *n* = 2 displays large peaks in the red curve, signifying strong local ordering and a high probability of finding another hydroxy oxygen atom at certain distances. Conversely, the peak of the blue curve for hydroxyl–ether oxygen atoms is lower, suggesting weaker interactions or a more dispersed distribution. This pattern aligns with the compact nature of the PEG dimer as seen in Fig. 4(a). In Fig. 7(b) and (c) for oligomers with *n* = 3 and *n* = 4, the peaks in both RDFs become sharper, reflecting an increasingly ordered atomic arrangement. The first peak in the red and blue curves is particularly sharp, highlighting a strong preference for specific interatomic distances. For longer chains, *n* = 5 to *n* = 7, as illustrated in Fig. 7(d) through Fig. 7(f), the peaks in the blue curve are more prominent compared to those in shorter oligomers, suggesting that the interactions between hydroxy and ether oxygen atoms intensify with increasing chain length.

The observed differences in structural properties between OPLS and GAFF force fields for PEG oligomers can be attributed to their development focuses. Notably, OPLS was specifically optimized for capturing molecular properties in liquid-phase environments. Consequently, this focus may result in suboptimal performance for transport properties like self-diffusion coefficients and shear viscosity, as these sensitive properties are highly dependent on the intricate molecular dynamics and the underlying potential energy landscape of the polymeric PEG system.

## Conclusion

In this study, molecular dynamics simulations have been used to investigate the thermophysical properties of ethylene glycol oligomers, focusing on oligomer sizes ranging *n* = 2–7. Utilizing the GAFF force field, we systematically calculated the density, self-diffusion coefficients, shear viscosity, and thermal conductivity of these oligomers.

The results revealed that the GAFF force field provides a robust framework for accurately predicting the behavior of ethylene glycol oligomers, aligning well with experimental data and outperforming other commonly used force fields. The GAFF force field demonstrates significant improvement in predicting self-diffusion and viscosity, with deviations with respect to experimental data below 10%. Other commonly used force fields, such as OPLS, CGenFF and GROMOS, report errors in the range 40–400%. The study reports, for the first time, thermal conductivities of polyethylene glycol (PEG) oligomers that are in good agreement with experimental data, demonstrating deviations below 20%. While our results demonstrate excellent agreement with experimental data at 328 K, it is essential to note that future studies should investigate the temperature dependence of PEG oligomer properties to fully understand the behavior of these molecules. Furthermore, investigations of charge modifications to the GAFF force field provided valuable insights into the influence of charge balance on the properties of PEG oligomers.

From the analyses of key measures such as average end-to-end distances, radii of gyration, and hydrogen bonding, correlations between oligomer size and thermophysical properties of PEG were highlighted. The analyses revealed a linear trend in the average end-to-end distances and radii of gyration as a function of oligomer size, indicating that larger oligomers adopt more extended conformations. This increase in size correlates with a higher molecular flexibility, as evidenced by the broadening distribution of end-to-end distances.

We found that subtle modifications to the charge distribution within the GAFF force field can significantly impact the bonding formation, which in turn affects the thermophysical properties of the PEG oligomers. This emphasizes the critical role of accurate charge modeling in molecular dynamics simulations and its implications for understanding the behavior of complex molecular systems such as PEG oligomers.

In summary, this study demonstrated an excellent performance of the GAFF force field for simulating the thermophysical properties of PEG oligomers, providing an alternative force field for such systems. While modifications to the atomic partial charges were discussed, a conclusion is that the original GAFF force field is preferred. Future research can build upon these findings by employing a combined AMBER/GAFF force field to study PEG–water interactions and explore the behavior of PEG oligomers in complex systems with other molecules.

## Data availability

The data supporting this article have been included as part of the ESI.†

## Conflicts of interest

There are no conflicts to declare.

## Supplementary Material

RA-014-D4RA04898A-s001
