# Supplementary material for: Thermophysical properties of polyethylene glycol oligomers via molecular dynamics simulations
Source: RSC Adv. 2024 Sep 3;14(38):28125–37. doi: 10.1039/d4ra04898a (PMC11369976; doi:10.1039/d4ra04898a)
Supplement: RA-014-D4RA04898A-s001 [file RA-014-D4RA04898A-s001.pdf]

Supplementary Information (SI) for RSC Advances.
This journal is © The Royal Society of Chemistry 2024

SUPPORTING INFORMATION: Thermophysical Properties of Polyethylene Glycol Oligomers via Molecular Dynamics Simulations

Thi H. Ho,[*,†,‡] Hien Duy Tong,[¶] Øivind Wilhelmsen,[§] and Thuat T. Trinh[*,§]

†*Laboratory for Computational Physics, Institute for Computational Science and Artificial Intelligence, Van Lang University, Ho Chi Minh City, Vietnam*

‡*Faculty of Mechanical - Electrical and Computer Engineering, School of Technology, Van Lang University, Ho Chi Minh City, Vietnam*

¶*Faculty of Engineering, Vietnamese-German University (VGU), Thu Dau Mot City, Binh Duong Province 75000, Vietnam*

§*Porelab, Department of Chemistry, Norwegian University of Science and Technology,Trondheim, Norway*

E-mail: thi.hohuynh@vlu.edu.vn; thuat.trinh@ntnu.no

Table S1: Force field coefficients for bond stretching.

Chemical group	Bonding Coeff.	K (kcal/Å^2)	r_0 (Å)
Hydroxyl ($-O-H$)	$O-H$	371.40	0.97
	$C-O$	316.70	1.42
Ether ($-CH_2-O-$)	$C-C$	300.90	1.54
	$C-H$	330.60	1.10
	$C-O$	308.60	1.43

Table S2: Force field coefficients for angle bending. Note that H_O represents the hydrogen atom in the hydroxyl group, H_C denotes the hydrogen atom on the ether linkage.

Chemical group	Angle Coeff.	K (kcal)	θ_0 (degree)
Hydroxyl ($-O-H$)	$C-C-O$	67.50	110.19
	$C-O-H_O$	47.40	107.26
	H_C-C-O	50.90	110.26
Ether ($-CH_2-O-$)	$C-C-H_C$	46.40	109.56
	$C-C-O$	68.00	107.97
	$C-O-C$	62.70	112.48
	H_C-C-H_C	39.20	108.46
	H_C-C-O	50.80	109.78

Table S3: Force field coefficients for dihedral torsion. Note that H_O represents the hydrogen atom in the hydroxyl group, H_C denotes the hydrogen atom on the ether linkage, O_H is the oxygen atom within the hydroxyl group, and O_E is the oxygen atom in the ether linkage.

Chemical group	Dihedral Coeff.	K (kcal)	n	d (degree)
Hydroxyl $(-O-H)$	$C-C-O-H_O$	0.25	1	0
	$H_C-C-C-O$	0.25	1	0
	$H_C-C-O-H_O$	0.17	3	0
	$O-C-C-O_E$	1.18	2	0
Ether $(-CH_2-O-)$	$C-C-O-C$	0.10	2	180
	$C-O-C-H_C$	0.38	3	0
	$H_C-C-C-H_C$	0.16	3	0
	$H_C-C-C-O$	0.25	1	0
	$O-C-C-O_H$	1.18	2	0
